# Redescription of three pinworms of the genus *Cephalobellus* Cobb, 1920 (Nematoda: Oxyuridomorpha: Thelastomatidae) from scarab beetle grubs from Hungary

**DOI:** 10.1007/s11230-023-10124-1

**Published:** 2024-02-19

**Authors:** Luca Eszter Balog, Mohammed Ahmed, Oleksandr Holovachov

**Affiliations:** 1 Doctoral School of Biology, Institute of Biology and Department of Systematic Zoology and Ecology, Eötvös Loránd University, Pázmány Péter sétány 1C, Budapest, 1117 Hungary; 2 Department of Zoology, Swedish Museum of Natural History, Box 50007, 104 05 Stockholm, Sweden; 3 Department of Evolution, Ecology and Behaviour, Institute of Infection, Veterinary and Ecological Sciences, University of Liverpool, Liverpool, L69 7AB UK

## Abstract

Larvae of European rose chafer *Cetonia aurata* (Linnaeus, 1758) and cockchafer *Melolontha* sp. beetles were collected in Hungary for parasitological study. Intestinal examination revealed the presence of three well-known thelastomatid nematodes belonging to the genus *Cephalobellus* Cobb, 1920. We report for the first-time *Cephalobellus cuspidatum* (Rudolphi, 1814) Leibersperger, 1960, *C. osmodermae* Leibersperger, 1960, and *C. potosiae* Leibersperger, 1960 in Hungary, all found in scarab beetle larvae. Due to incomplete original descriptions, a comprehensive redescription with detailed morphological data is presented. Additionally, an identification key for closely related *Cephalobellus*, *Thelastoma* and *Severianoia* species infesting scarab beetles worldwide is provided. Newly generated 18S and 28S rDNA gene sequences of *C. osmodermae* place it as one of the early branches within Thelastomatidae.

## Introduction

The family Thelastomatidae (Nematoda: Oxyuridomorpha: Thelastomatidae) proposed by Travassos in 1929 currently comprises 1 subfamily, 42 genera and 268 species (Hodda, 2022). Thelastomatid pinworms are obligate endosymbiotic nematodes that occur in the intestinal tract of the invertebrate host and feed on bacterial microbiomes. These species have a worldwide distribution, but their diversity and biology are poorly known. The classification of the genera within the family Thelastomatidae is generally based on morphology, with limited molecular support and likely not reflecting true evolutionary relationships. The current classification of Thelastomatidae categorizes it as a monophyletic group. However, the phylogenetic analyses of ribosomal genes suggest that this group is paraphyletic and poorly resolved (Carreno, 2014; Garduño Montes de Oca & Oceguera-Figueroa, 2020; Zhang et al, 2021) due to existence of many insufficiently known genera and species, along with significant gaps in molecular data.

The genus *Cephalobellus* Cobb, 1920 is the second most species-rich, after *Thelastoma* Leidy, 1849, within the family Thelastomatidae. The first descriptions of thelastomatid pinworm from arthropods was *Cephalobellus cuspidatum* (Rudolphi, 1814) Leibersperger, 1960, originally named *Ascaris cuspidata* Rudolphi, 1814. This species was isolated from the intestine of the rhinoceros beetle’s grub *Oryctes nasicornis* (Coleoptera: Scarabaeidae). To date, 34 different species from this genus have been described, isolated from the hindguts of beetle larvae (Coleoptera), crane fly larvae (Tipulidae), adult millipedes (Diplopoda), adult cockroaches (Blattaria), and adults and larvae of flies (Diptera) (Adamson & van Waerebeke, 1992; Camino & Reboredo, 2000, 2005; Jex et al., 2006b; Hodda, 2022). On the other hand, there is very little phylogenetic and ecological data available for these species, besides what was included in the original descriptions. No data about the co-infection of the same host and limited information about phylogenetic relationships of these species were available until now.

The differences between the genera *Cephalobellus* and *Thelastoma* and the identification of species within each is a challenging task. Recent species descriptions within these genera primarily focus on interspecific differences within particular genera, and do not discuss similarities or differences between *Cephalobellus* and *Thelastoma* (Carreno, 2007; Carreno et al, 2018; Jex et al., 2006b; Orsini et al., 2018). With more species described the differences between *Cephalobellus* and *Thelastoma* became less clear. For example, Liebersperger (1960) proposed that a filiform tail is characteristic for the genus *Thelastoma* genus, while a non-filiform tail is typical for *Cephalobellus*. However, many species with long filiform tails have been reassigned to different genera from *Thelastoma* since Liebersperger's original descriptions in 1960.

Our attempt to build the identification key revealed that some described species cannot be clearly delineated from each other based on morphological characters and may possibly be synonymous, especially if they inhabit the same host. On the other hand, some species known from distantly related hosts (Diplopoda versus Scarabaeidae) have been synonimised in the literature (Liebersperger, 1960) without much scrutiny – it is not unlikely that these are morphologically similar but ecologically and genetically different cryptic species – a question that cannot be answered without in-depth analysis of their genomes and reproduction. All species redescribed here are known only from their original descriptions; there is no information on their geographic variability and no molecular data as well. Furthermore, thorough morphological redescriptions of species, accompanied with details on host specificity and geographic distribution, will clarify the systematics and biology of these common but insufficiently studied parasites. Moreover, several species of the genus *Severianoia* (Schwenk, 1926) Travassos, 1929 are found in scarab beetle larvae and are morphologically similar to *Cephalobellus* and *Thelastoma –* they were also included in the key,

Certain scarab beetles (Coleoptera: Scarabaeoidea) can be significant threats as agricultural pests, emphasizing the economic importance of studying and examining their parasites (Huiting et al., 2006). However, the knowledge about the nematode parasites associated with these scarab beetles, including members of the family Thelastomatoidea, is still limited. It is noteworthy that no thelastomatid pinworms have been identified in Hungary at species level before (Balog et al., 2022a, 2022b; Balog & Török, 2019).

This study presents both ecological data on the co-infection and the redescriptions of three species found in scarab grubs in Hungary: *Cephalobellus cuspidatum*, *C. osmodermae* Leibersperger, 1960 and *C. potosiae* Leibersperger, 1960 using light and scanning electron microscopy. The relationships between closely related genera *Cephalobellus, Thelastoma* and *Severianoia* are discussed based on molecular phylogenies that include newly sequenced *C. osmodermae*.

## Materials and Methods

*Host collection and nematode isolation*. Numerous European rose chafer *Cetonia aurata* (Scarabaeidae: Cetoniinae) and cockchafer *Melolontha* sp. (Scarabaeidae: Melolonthinae) beetles were collected at the larval stage and identified using morphological characters by Professor Ottó Merkl, an entomologist affiliated with the Hungarian National Museum of Natural History. Insect grubs were isolated from soil and compost from 10 different localities of Hungary (Table 1) from September of 2017 to May of 2023. The 128 collected beetles were brought alive to the laboratory and stored in a refrigerator until dissection. The grubs were decapitated and dissected under BTC STM3c stereomicroscope. The intestine of the host was isolated and investigated separately under stereomicroscope. The nematodes found in the host’s intestine were fixed as described below.

**Table 1:** Prevalence and intensity of *Cephalobellus cuspidatum* (Rudolphi, 1814) Leibersperger, 1960, *C. osmodermae* Leibersperger, 1960 and *C. potosiae* Leibersperger, 1960

Locality	Host species	Number of hosts examined	*C. cuspidatum* infection			*C. osmodermae* infection			*C. potosiae* infection		
			n	prevalence	intensity^1^	n	prevalence	intensity^1^	n	prevalence	intensity^1^
Pomáz, compost	*Cetonia* sp.	17	11	65%	12.7 (2-30)	9	53%	10.4 (2-30)	0	–	–
Börzsöny, forest soil	*Cetonia* sp.	6	2	33%	2.5 (2-3)	1	17%	2	0	–	–
Budaörs; compost	*Cetonia* sp.	9	0	–	–	9	100%	25.3 (4-45)	0	–	–
Budapest district 23th; compost A	*Cetonia* sp.	14	0	–	–	0	–	–	0	–	–
Budapest district 23th; compost B	*Cetonia* sp.	7	0	–	–	0	–	–	0	–	–
Budapest district 8th; garden soil	*Melolontha* sp.	8	3	38%	8.7 (2-15)	1	13%	8	1	13%	5
Budapest district 23th; garden soil	*Melolontha* sp.	33	10	30%	6.3 (2-17)	3	9%	5 (2-10)	2	6%	4.5 (4-5)
Alsó-Göd, garden soil	*Melolontha* sp.	12	2	17%	8.5 (5-12)	3	25%	9.6 (3-18)	0	–	–
Komárom; garden soil	*Melolontha* sp.	14	–	–	–	4	29%	5.25 (2-10)	0	–	–
Gödöllő; garden soil	*Melolontha* sp.	8	–	–	–	6	75%	9.5 (3-21)	0	–	–
		**Mean**		**37%**	**7.7 (2-30)**		**40%**	**9.4 (2-45)**		**9%**	**4.8 (4-5)**

^1^Infections’ mean and range

*Morphological characterization of nematodes*. For light microscopic examination, the isolated nematodes were fixed in FAA solution (20 parts 90% ethanol, 6 parts 40% formalin, 1 part glacial acetic acid, 40 parts water). After fixation, the samples were dehydrated in glycerol–ethanol mixture (9 parts 90% alcohol, 1 part glycerol) (Andrássy & Farkas, 1988) and mounted on permanent glass slides using paraffin as a support for the cover slip. Morphological studies were carried out with Zeiss Axioskop 2 microscope, using transmitted light and DIC illumination and an Olympus Color View digital camera with DP-Soft software and Nikon Eclipse 80i or Nikon Eclipse Ni microscopes, both of which are equipped with DIC illumination and Sony a6400 camera. Several nematodes were prepared for scanning electron microscopy (SEM) by fixing in FAA and dehydration through graded ethanol series (25 to 99.8%). The specimens were then soaked in amyl-acetate and critical-point dried. The dried nematodes were coated with gold and observed with a scanning electron microscope (SEM Hitachi 2360N). Identification of nematodes was done by comparing recent specimens with the information provided in the original descriptions and redescription of species from the genera *Cephalobellus* and *Thelastoma* (summarised in Leibersperger, 1960; Jex et al., 2006a).

*Molecular analysis*. For molecular characterization the nematodes were fixed in 96% ethanol. DNA extraction was performed by a modified HotSHOT method (Truett et al., 2018) on multiple specimens of *Cephalobellus osmodermae* from different localities. Before the extraction, individual nematodes were mounted on temporary slide and several photographs were taken from each of them. The nematodes were placed individually in 0.2 ml PCR tubes containing 25 μl lysis buffer (25 mM NaOH, 0.2 mM disodium EDTA and a pH of 8) and incubated on a microplate shaker at 95°C and 300 rpm for 30 minutes. The lysed samples were placed on ice for 5 minutes and 25 μl neutralizing buffer was added (40 mMTris-HCl and a pH of 5 prepared by dissolving Tris-HCl). The approximately 1800 bp region of the 18S rRNA gene was amplified as two overlapping fragments using the primer sets 988F (5ʹ-CTCAAAGATTAAGCCATGC-3ʹ) and 1912R (5ʹ-TTTACGGTCAGAACTAGGG-3ʹ) for the first fragment and 1813F (5ʹ-CTGCGTGAGAGGTGAAAT-3ʹ) and 2646R (5ʹ-GCTACCT GTTACGACTTTT-3ʹ) for the second fragment (Holterman et al., 2006). The D2-D3 segment of the 28S rDNA region was amplified using the primers D2Af (5ʹ-ACAAGTACCGTGAGGGAAAGTTG-3ʹ) and D3Br (5ʹ-TCGGAAGGAACCAGCTACTA-3ʹ) (Nunn, 1992). The polymerase chain reaction (PCR) was performed in a 25 μl reaction mix DNA using Taq DNA Polymerase (Thermo Scientific™ EP0404), containing 5 µl template DNA, 1-1 μl of both 10 μM primers, 5 μl 10 mM dNTP (Thermo Scientific™ R0192). The PCR conditions of both fragments of the 18S rDNA region were 5 min at 95 °C; 5 cycles of (94 °C for 30 sec, 45°C for 30 s and at 72 °C for 30 sec); 35 cycles of (94 °C for 30 sec, 54 °C newly obtained sequences were deposited in the NCBI GenBa for 30 s and 72 °C for 30 s); and a final extension for 5 min at 72 °C. The PCR condition of the D2-D3 segment of the 28S rDNA region were as follows: 4 min at 94 °C; 35 cycles of (94 °C for 60 s, 54 °C for 90 s and 72 °C for 2 min); final extension for 10 min at 72 °C. Enzymatic PCR clean-up was performed on the PCR product using Thermo Scientific™ GeneJET PCR Purification Kit. The purified PCR products were sent out to Eurofins BIOMI (Gödöllő, Hungary) and Szegedi Biológiai Kutatóközpont (Szeged, Hungary) for sequencing. Each fragment of 18S and 28S rDNA was sequenced in both directions using the forward and reverse PCR primers. Trimming and merging of sequences were performed using a custom script. For each fragment, the script first converts each sequence from ab1 format to fastq using *seqret* (Rice et al., 2000). The fastq sequences were then quality trimmed using *seqtk* (https://github.com/lh3/seqtk) with quality threshold set to 0.01 (Q20). The reverse sequence is then reverse complemented before being merged with the forward sequence using *merger* (Rice et al., 2000). The full length 18S rDNA was obtained by merging the two individual fragments.

*Phylogenetic analysis*. Four datasets were created by combining recent sequences with GenBank sequences of Thelastomatidae using AliView (Larsson, 2014) built-in MUSCLE algorithm (muscle3.8.31_i86darwin64): (1) alignment using nearly complete 18S rDNA sequences; (2) alignment combining nearly complete and partial 18S rDNA sequences; (3) alignment using D2D3 fragment of 28S rDNA sequences; (4) concatenated alignment combining nearly complete and partial 18S rDNA with D2D3 fragment of 28S rDNA sequences for those species which had both available. Since the main goal of the analysis was to evaluate relationships between the genera *Cephalobellus* and *Thelastoma,* only representatives of the family Thelastomatidae were included in the analysis, with the exception of two species of *Pseudonymus* Diesing, 1857 (Pseudonymidae) which were used as an outgroup for all analyses based on Holterman et al. (2019). Phylogenetic hypotheses were inferred independently for each dataset/alignment using IQTree (Minh et al., 2020) using built in ModelFinder (Kalyaanamoorthy, 2017) to infer best fit substitution model with the following command: ./iqtree2 -s ./filename.fas -st DNA -m MFP -b 1000 -T AUTO. Cladograms were edited in TreeViewer (Bianchini & Sánchez-Baracaldo, 2023). The newly obtained sequences were deposited in the NCBI GenBank with accession numbers OR514711-OR514716; OR526602-OR526603.

### **Thelastomatidae** Travassos, 1929

***Cephalobellus*** Cobb, 1920

*Genus diagnosis* emended after Adamson and van Waerebeke (1992): Females defined as having a cephalic extremity formed by a circumoral labial annule and a larger first body annule. Pharynx composed of three parts: corpus, isthmus, and basal bulb. Corpus cylindrical. Isthmus a short construction between corpus and bulb. Basal bulb ovoid and spherical with valvular apparatus. Reproductive system didelphic. Vulval slit located near midbody. Vagina directed anteriorly. Each uteri connects with a long and flexed ovary. Eggs elongate. Tail conical, attenuate, filiform or cupola-shaped with spike-like projection. Males differ from females in size and body shape. Male cephalic region formed by short annules. Caudal extremity conical to attenuate. Spicule present. Two pairs of pre-anal (or ad-anal) and two pairs of postanal papillae present.

### ***Cephalobellus cuspidatum*** (Rudolphi, 1814) Leibersperger, 1960

*Synonyms: Ascaris cuspidata* Rudolphi, 1814; *Oxyuris nasicornis* Dugès, 1826; *Anguillula cuspidata* (Rudolphi, 1814) Diesing, 1851; *Thelastoma cuspidatum* (Rudolphi, 1814) Théodoridès, 1955; *Isacis cuspidata* (Rudolphi, 1814) Diesing, 1860.

Type specimens: syntype, 473-E, Museum für Naturkunde Berlin.

*Type host: Oryctes nasicornis* (Rudolphi, 1814)*.*

*Known hosts*: *Anomala dubia* var. *aenea* and *Anoxia* sp. (Diesing, 1851).

*Current host: Cetonia aurata* (Scarabaeidae; Coleoptera).

*Type locality:* Germany (Rudolphi, 1814).

*Known localities*: Germany (Leibersperger, 1960), France (Dugès, 1826; Diesing, 1851).

*Current locality*: Hungary.

*Location in the host*: Intestine.

*Prevalence and intensity*: average prevalence of 37% (17–65%), five of the 10 investigated populations were infected; average intensity of 7.7 (2–30) (Table 1). Found with *Cephalobellus osmodermae* in three *Cetonia aurata* host individuals; found with *Cephalobellus potosiae* in one *Melolontha* sp. individual; found with *C. osmodermae* and *C. potosiae* in one *Melolontha* sp. individual; found with *Reiterina typica* (Stefański, 1922) Sudhaus, 2011 in two *Melolontha* sp. host individuals.

*Material examined*: 30 female specimens, deposited in the invertebrate collection of the Swedish Museum of Natural History, Stockholm (slide accession numbers SMNH219625-SMNH219627).

### Redescription

**Fig. 1.**
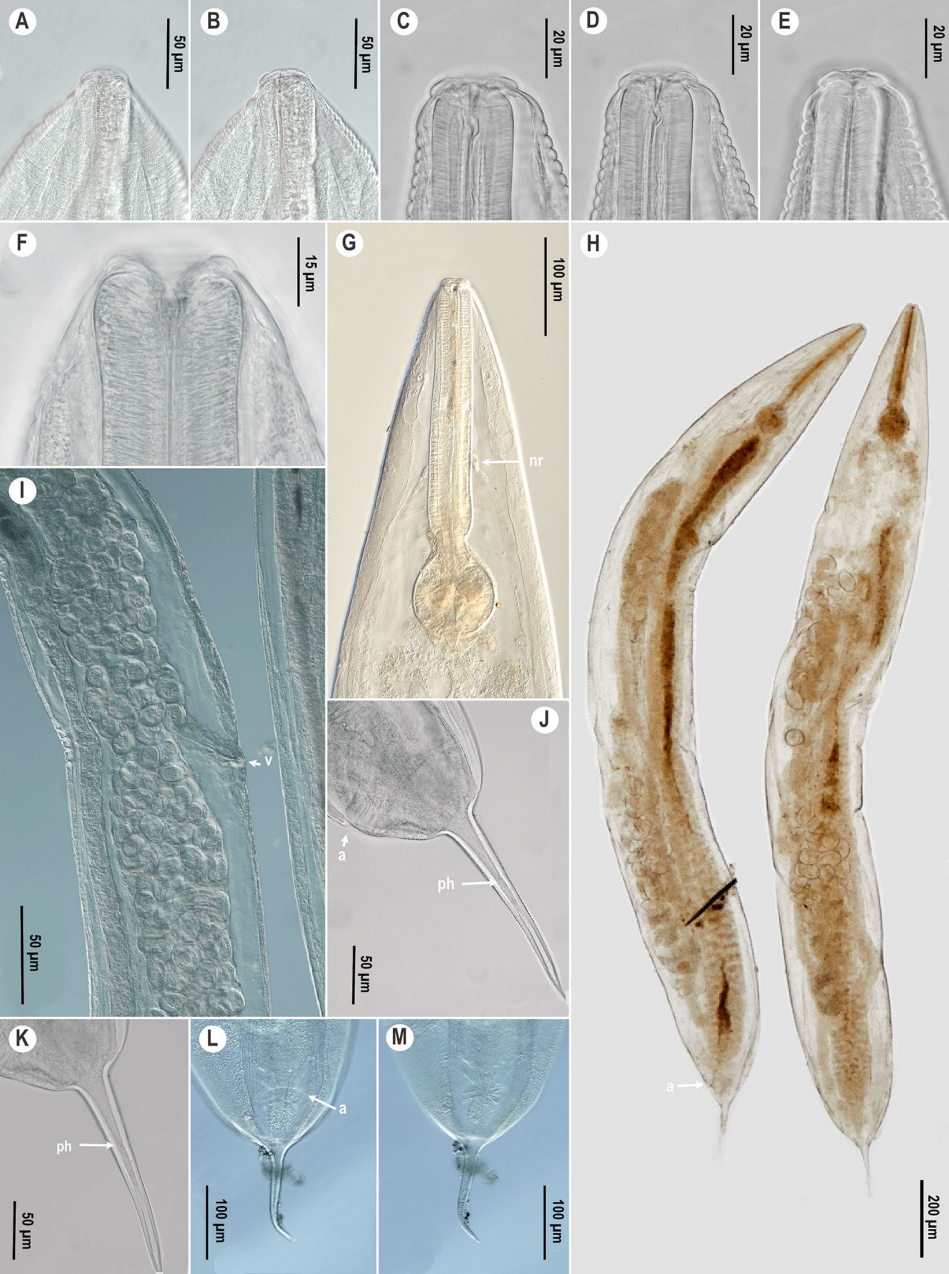
*Cephalobellus cuspidatum* (Rudolphi, 1814) Leibersperger, 1960 female: A-F, Anterior body end and stomatal region of different individuals in different focal planes, showing variability of stoma and labial region morphology; G, Pharyngeal region, lateral view; H, Entire body of different individuals, sublateral view; I, Vulval region, lateral view; J-M, Tail with one pair of phasmids in different view (J-K, in lateral view, L-M, ventral view). *Abbreviations*: nr: nerve ring; v: vulva; a: anal opening; ph: phasmid.

**Fig. 2.**
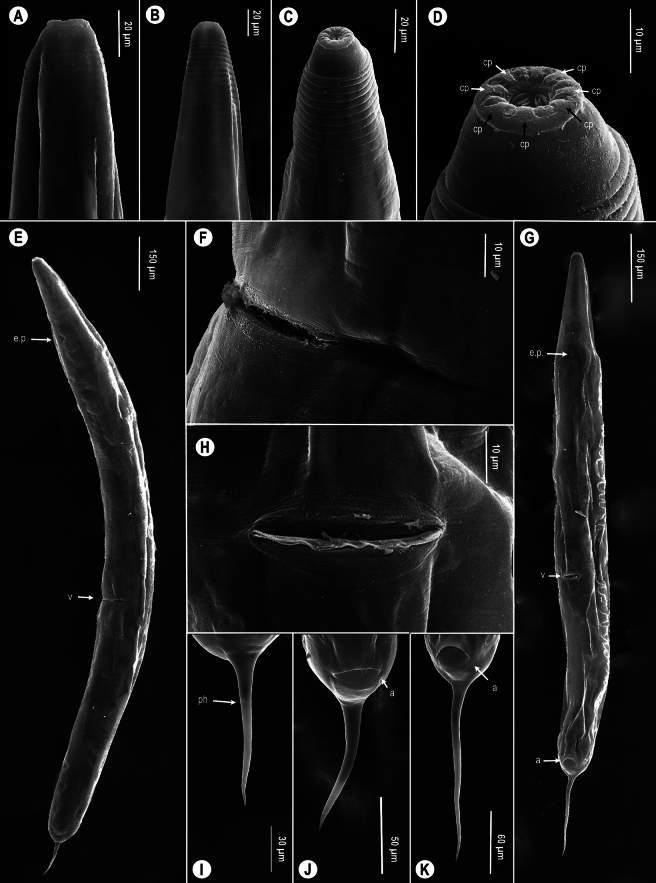
*Cephalobellus cuspidatum* (Rudolphi, 1814) Leibersperger, 1960 (A-B, E-F, I-J) and *Cephalobellus osmodermae* Leibersperger, 1960 (C-D, G-H, K) scanning electron micrographs of females. A-D, Cephalic region; E, G, Entire body, subventral view; F, H, Vulva; I-K, Tail. *Abbreviations*: cp: cephalic papillae; ep: excretory pore; v: vulva; a: anal opening; ph: phasmid.

*Female*. (Figures 1, 2A-B, E-F, I-J, Table 2.) Body spindle-shaped (Figs 1H, 2E), reaching maximum body width at vulval region. Cuticle clearly transversely annulated. Annules along entire pharyngeal region similar in size. In posterior quarter of body annules are decreasing in size. Lateral alae absent. Labial annule about 7 µm height, 33 µm width, followed by a much larger first body annule, extending about 19 µm height and 54 µm width (Figs 1C-D, 2A-B). Labial lobes not prominent, eight cephalic papillae present. Amphids not visible under the light microscope and under SEM. Females with low stoma. Soma opening triradiate, surrounded by hexagonal lining of cuticle (Fig. 1F). Nerve ring located approximately in middle region of pharynx (Fig. 1G). Excretory pore easily detectable, located posterior to base of basal bulb, at 14–21% of total body length. Vulval slit not prominent and located approximately at midbody (Fig. 2F). Uterus containing numerous oval eggs. Eggs 1.5 times as long as wide (Fig. 1I). Egg shell without distinct ornamentation. Tail region dome-shaped ending in a short conical spike-like projection that is 1.5–4 times longer than dome (Figs 1J-M, 2I-J). Phasmids present, at 35–40% of the tail spike or at 60% of total tail length (Fig. 1J-M).

**Table 2.** Morphometrics of *Cephalobellus cuspidatum* (Rudolphi, 1814) Leibersperger, 1960, *C. osmodermae* Leibersperger, 1960 and *C. potosiae* Leibersperger, 1960: measurements in μm and in the form: mean ± s.d. (range) where available

	*Cephalobellus cuspidatum*		*Cephalobellus osmodermae*				*Cephalobellus potosiae*		
	present study	Jex et al., 2006a, 2006b	present study		Leibersperger, 1960		present study	Leibersperger, 1960	
	*Female*	*Female*	*Male*	*Female*	*Male*	*Female*	*Female*	*Male*	*Female*
n	12		10	10			6		
L	3640 ± 751 (2471–4578)	5700	836 ± 242 (199–1100)	1891 ± 186 (1594–2098)	660–810	1620–2230	4907 ± 789 (4406–6301)	540–1560	2920–7360
a	10.6 ± 1.2 (8–11.9)	8.10	13.4 ± 4.9 (3.3–20)	10.4 ± 3.5 (2.1–14.4)	―	―	11.3 ± 2.6 (9.6–16)	―	19.5–36.8
b	7.8 ± 1 (6.6–9.5)	9.50	4.1 ± 1.1 (1–5.2)	5.4 ± 0.4 (4.9–6.2)	―	5.3–6	11 ± 1.2 (10.2–13.1)	―	6.1–10.5
c	17.7 ± 2.6 (14.4–22.1)	11.40	15.1 ± 4.7 (4.7–21.3)	6.5 ± 1 (4.9–8.4)	―	6.2–7.2	32.4 ± 6.4 (24.3–39.7)	―	19.5–36.8
c'	1.5 ± 0.2 (1.2–1.8)	―	3 ± 1.2 (1–4.4)	3.9 ± 0.7 (2.8–4.9)	―	―	0.8 ± 0.1 (0.7–1)	―	―
T or V^1^	53 ± 6 (42–62)	46	45 ± 35 (30–138)	52 ± 4 (44–56)	―	52–53	47 ± 2 (44–49)	―	53–57
Height of labial annule	7 ± 1.4 (4.4–9)	―	―	5.7 ± 0.8 (4.8–7.2)	―	―	6.6 ± 1.1 (5.1–7.8)	―	―
Width of labial annule	33.1 ± 2.8 (29.5–38)	―	―	30.5 ± 2.1 (27.6–33.7)	―	―	33.5 ± 1.9 (31.4–35.7)	―	―
Height of first body annule	18.8 ± 2.9 (14.5–22)	―	―	15.1 ± 2.2 (11.8–18.2)	―	―	17.3 ± 3.4 (12–21.2)	―	―
Width of first body annule	53.8 ± 7.2 (43.6–63.8)	―	―	42.3 ± 1.9 (39.2–44.7)	―	―	58.2 ± 8.3 (46.2–69)	―	―
Height of second body annule	5 ± 1.6 (3–8)	―	―	4.4 ± 0.4 (3.9–5.1)	―	―	5.6 ± 0.9 (4.3–6.7)	―	―
Width of second body annule	59.7 ± 8.7 (47.6–70)	―	―	46.4 ± 5.9 (39.5–61.5)	―	―	64 ± 8.4 (52–75)	―	―
Stoma depth (entire)	14.4 ± 1.5 (12–16)	―	7.4 ± 1.2 (6.3–10)	12.5 ± 2.1 (9.7–17)	―	―	14.5 ± 2.2 (12–27.3)	―	―
Cheilostom length	4.9 ± 1.4 (3–7.2)	―	2.9 ± 0.7 (1.7–3.7)	4.2 ± 0.9 (3–5.4)	―	―	4.2 ± 1 (3.3–5.6)	―	―
Cheilostom width	8.8 ± 2.2 (5.9–11.5)	―	2.4 ± 1 (0.9–4)	9.4 ± 1.8 (6–11.6)	―	―	9.5 ± 1.6 (7.8–11.2)	―	―
Pharyngostom length	9.6 ± 0.8 (8.1–10.8)	―	4.4 ± 1.2 (3.6–6.7)	8.2 ± 2.4 (5.6–14)	―	―	9.6 ± 0.9 (8.9–11)	―	―
Phatyngostom width	5.8 ± 3 (2.3–11.3)	―	1.7 ± 0.6 (0.9–2.3)	3 ± 0.4 (2.4–3.5)	―	―	6 ± 3.3 (3–9.7)	―	―
Corpus length	335 ± 44.5 (268–401)	―	139 ± 15 (110–160)	237 ± 32.2 (165–277)	138–152	220–250	305 ± 38 (236–354)	100–182	370–520
Corpus diam.	44.8 ± 5.2 (38–56.4)	―	17.8 ± 1.9 (15.5–21.5)	39 ± 2.7 (35–44)	13–17	24–31	51.2 ± 5.2 (40.6–54)	12–17	24–42
Isthmus length	19.4 ± 2.8 (13.7–24)	―	13.4 ± 3.3 (10–19)	19.7 ± 2.5 (15.4–23.4)	―	―	21.2 ± 5.2 (15.4–27)	―	―
Basal bulb length	96.2 ± 11.3 (80–114)	―	45.9 ± 3.1 (41.4–51.6)	77.4 ± 6.6 (67–90.5)	43	―	97 ± 5.2 (92.7–104.3)	28–67	103–151
Basal bulb diam.	94.4 ± 11 (78–112)	―	40.6 ± 3.5 (36.2–46.6)	73.9 ± 6.2 (66.7–84)	38–39	―	98.6 ± 7.6 (90–110.3)	31–42	106–151
Pharyngeal region length^2^	462 ± 53.2 (376–537)	―	204 ± 16.7 (174–229)	348 ± 29.4 (312–401)	―	―	446 ± 19.5 (432.9–480)	―	―
Nerve ring from anterior end	263 ± 66.6 (207–458)	―	120 ± 13.4 (107–147)	173 ± 12.9 (155–195)	116	158–208	245 ± 11.9 (235–258)	158–170	225–360
Excretory pore from anterior end	693 ± 91.1 (601–889)	―	235 ± 20.9 (220–274)	345 ± 38.5 (249–394)	260	330–475	―	247-490	670–1450
Vulva from anterior end	1888 ± 283 (1475–2486)	―	―	992 ± 124 (802–1150)	―	―	2052 ± 133 (2029–2312)	―	―
Vulva from posterior end	1785 ± 527 (1011–2678)	―	―	904 ± 109 (726–1118)	―	790–1040	1225 ± 60 (2262–2395)	―	1260–3490
Vulval / maximum body diam.	338 ± 59.2 (227–430)	700	66 ± 21.5 (47.5–114)	171 ± 39.6 (113–215)	50–62	140–180	424 ± 42.5 (350–459)	38–115	238–440
Egg length	65.3 ± 3.9 (61–75)	―	―	72 ± 4.9 (66–80)	―	77–90	68.5 ± 6.5 (59–77)	―	75–90
Egg diam.	43.6 ± 3.3 (40–52)	―	―	43.4 ± 3.9 (37–50)	―	43–50	47.7 ± 5.9 (39–56)	―	31–36
Total spicule length (chord)^3^	―	―	47.2 ± 4.5 (39.9–52.4)	―	44–45	―	―	31–41	―
Total spicule length (arc)^4^	―	―	47.9 ± 4.6 (41–53.9)	―	―	―	―	―	―
Cloacal or anal body diam.	141 ± 32.6 (100–217)	―	14.7 ± 2.1 (12–18)	77.2 ± 13.4 (57–100)	―	―	―	―	―
Tail length	207 ± 40.4 (152–280)	―	57.7 ± 17.4 (42–100)	293 ± 38.2 (250–380)	48–84	230–360	145 ± 41.4 (90–185)	65–99	150–200
Length of dome- shaped part of tail	62.5 ± 12.1 (42–89.4)	―	19.3 ± 6.9 (13–36)	36.4 ± 10.3 (20–60)	―	―	71.2 ± 20.3 (44.6–91)	―	―
Length of distal tail spike	144 ± 33.4 (97–212)	―	39.3 ± 13.9 (28–74)	258 ± 32.9 (214–325)	―	―	76.7 ± 20.9 (45.6–96)	―	―
Spike / Dome ratio	2.4 ± 0.6 (1.7–3.8)	―	1.7 ± 0.6 (0.6–2.5)	7.5 ± 2.3 (5.4–13.3)	―	―	―	―	―
Phasmid from anus	127 ± 10.4 (116–137)	―	―	―	―	―	―	―	―

^1^Anterior body end to the vulva; Gonad length including reflex part and vas deferens; ^2^From anterior end; ^3^Length of straight line from anterior tip of manubrium to distal tip of spicule; ^4^Curved median line from anterior tip of manubrium to distal tip of spicule

*Male.* Not found.

### ***Cephalobellus osmodermae*** Leibersperger, 1960

*Type specimens*: location unknown.

*Type hosts*: *Osmoderma eremita* and *Potosia aeruginosa* (Leibersperger, 1960)

*Current host: Cetonia aurata* (Scarabaeidae; Coleoptera).

*Type locality*: Germany (Leibersperger, 1960).

*Current locality*: Hungary

*Location in the host*: Intestine

*Prevalence and intensity*: average prevalence of 40% (9–100%), eight of the 10 investigated populations were infected; average intensity of 9.4 (2–45) (Table 1). Found with *C. cuspidatum* in three *Cetonia aurata* host individuals; found with *C. cuspidatum* and *C. potosiae* in one *Melolontha* sp. individual; found with *Reiterina typica* (Stefański, 1922) Sudhaus, 2011 in three *Melolontha* sp. host individuals.

*Material examined*: 30 female and 20 male specimens, deposited in the invertebrate collection of the Swedish Museum of Natural History, Stockholm (slide accession numbers SMNH219628-SMNH219632).

### Redescription

**Fig. 3.**
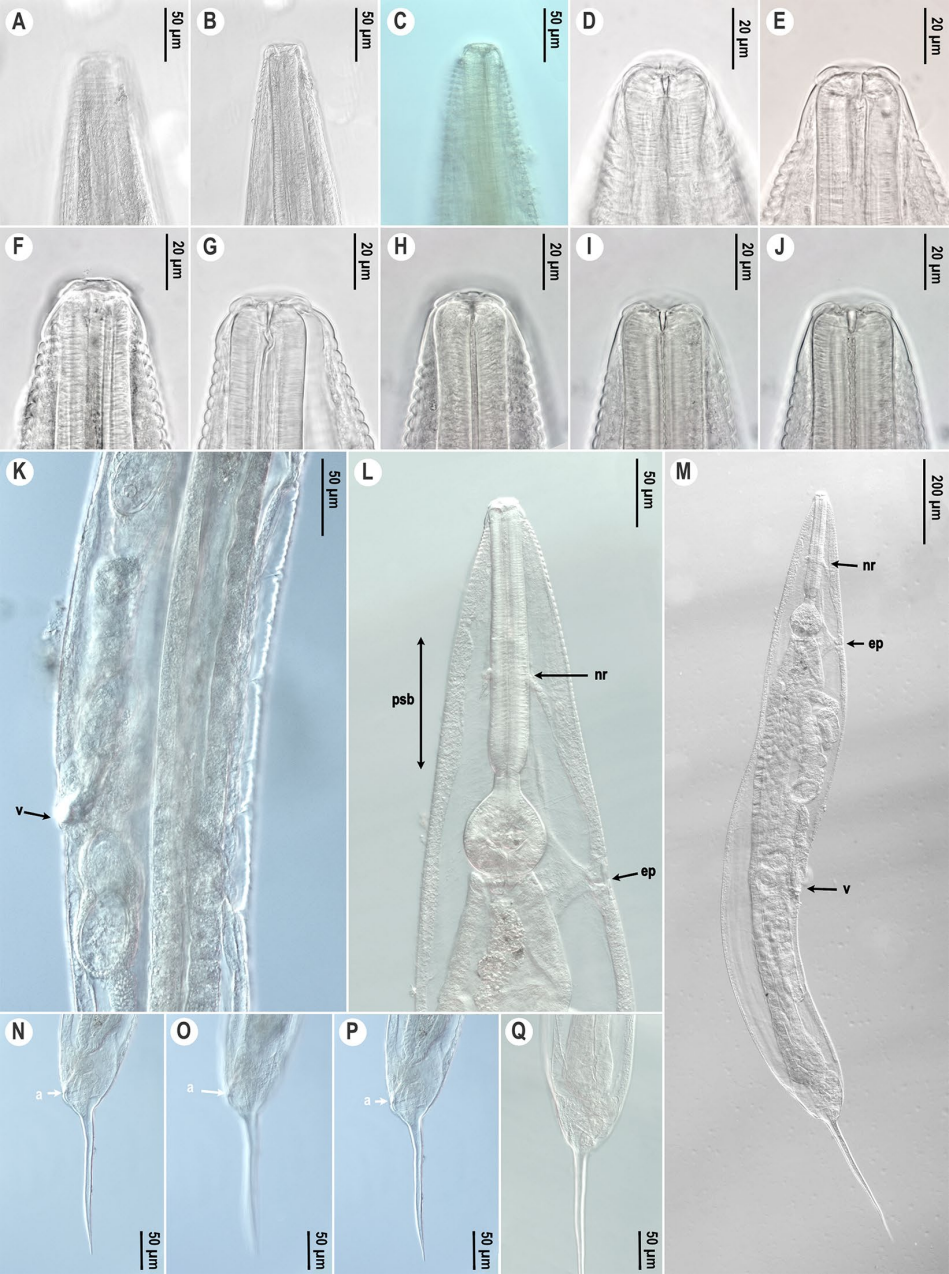
*Cephalobellus osmodermae* Leibersperger, 1960 female: A-J, Anterior body end of different individuals in different focal planes, showing variability of stoma and labial region morphology; K, Vulval region, lateral view; L, Pharyngeal region, corpus with pseudobulb, isthmus, and basal bulb, sublateral view; M, Entire body, lateral view, N-Q, Tail in different focal planes (N-L, lateral view; Q, ventral view). *Abbreviations*: nr: nerve ring; ep: excretory pore; psb, pseudobulb; v: vulva; a: anal opening.

*Female.* (Figures 2C-D, G-H, K and 3, Table 2) Body spindle-shaped (Fig. 3M), reaching maximum body width at vulval region. Cuticle transversely striated and clearly annulated. Annules larger and more prominent from anterior end to level of middle of corpus (about 12–15 annules) (Figs 2C, 3E). Size of annules desceases from there to behind anus, but clearly detectable. Lateral ale absent. Labial annule 6 µm height, 31 µm width, followed by longer, about 15 µm height, 42 µm width first body annule (Figs 2C-D, 3D-J). Labial lobes not well-developed, eight fused labiopapillae present (Fig. 2D). Amphids not easily detectable under light microscope. Opening of low stoma triradiate, surrounded by hexagonal lining of cuticle (Fig. 2D). Nerve ring located approximately at middle region of pharynx (Fig. 3L). Corpus cylindrical, pseudobulb present in second half of corpus. Excretory pore at level or a little posterior of base of basal bulb, at 14–22% of total body length. Uterus containing mostly 10–20, but recently more oval eggs. Eggs 1.5 times as long as wide. Egg shell without distinct ornamentation. Tail region dome-shaped ending in a short conical projection. Tail spike about 5–13 times longer than dome (Figs 2K, 3N-Q). Phasmid not seen.

**Fig. 4.**
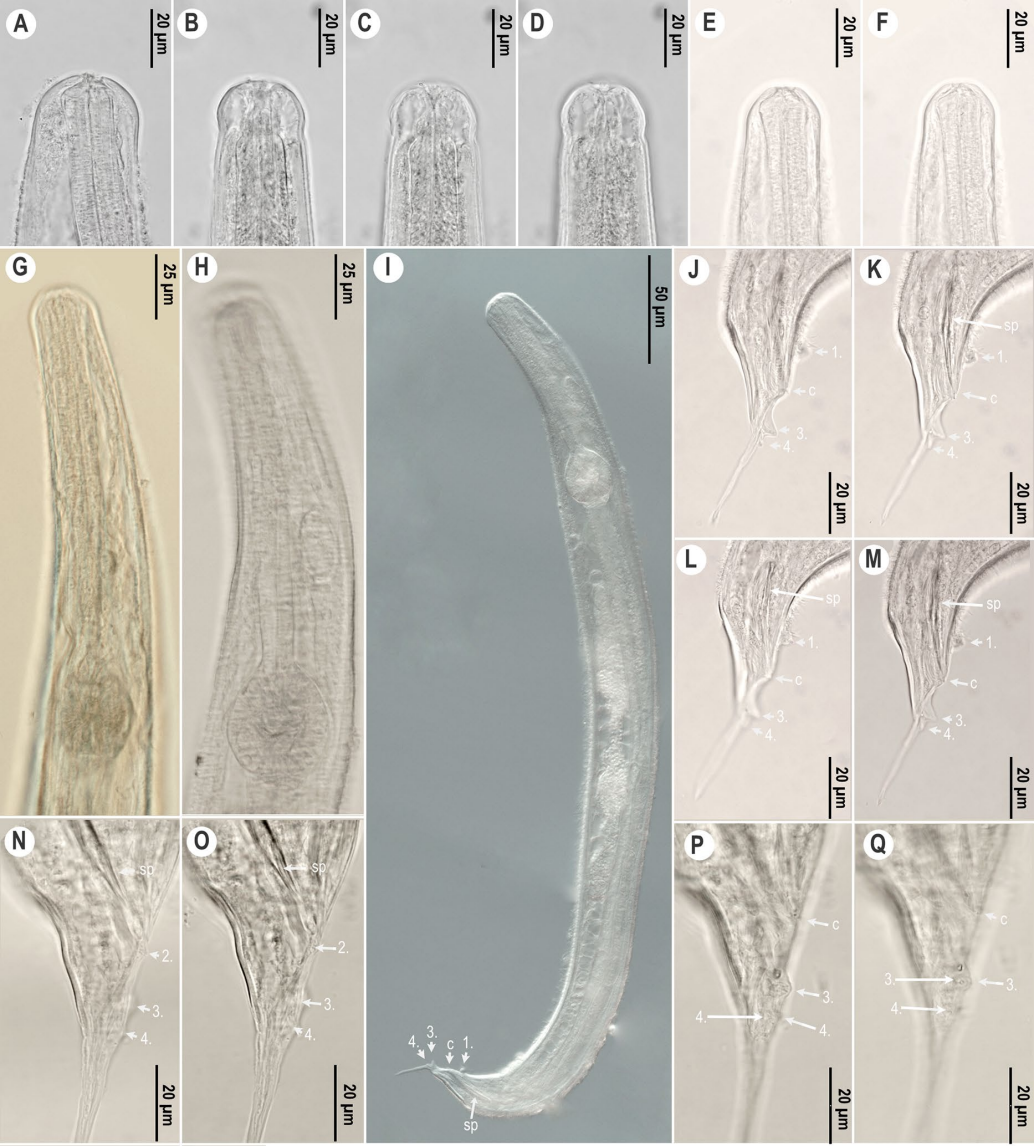
*Cephalobellus osmodermae* Leibersperger, 1960 male: A-F, Anterior body end of different individuals in different focal planes, showing variability of stoma and labial region morphology; G-H, Pharyngeal region, lateral view; I, Entire body, lateral view; J-Q, Posterior end, tail, cloacal opening, spicule, one pair precloacal, one pair adcloacal and two pairs postcloacal papillae (J-M, lateral view; N-Q, subventral view). *Abbreviations*: sp: spicule, c: cloacal opening, 1-4: numbers of genital papillae pairs.

*Male.* (Figure 4, Table 2) Male body shorter than females. Body thin, posterior part curved, maximum width at middle of body. Cuticle with regular transverse striations with annules increasing gradually and becoming more prominent in middle of body (Fig. 4I). Cuticle along anterior end is smooth, annulation absent (Fig. 4A-F). Lateral ale absent. Labial ring not set off from rest of body. Stoma very short, opening and labiopapillae not visible (Fig. 4A). Nerve ring located approximately at middle region of pharynx. Corpus cylindrical, narrows slightly to connect with a short cylindrical isthmus. Basal bulb ovoid with valvular apparatus (Fig. 4H). Excretory pore at level of base of basal bulb, at 24–34% of total body length. Testis long, extends from cloacal aperture, folding on itself. Genital cone prominent, cuticle thicker on its dorsal side, expands about 3.5 µm. Genital cone and tail region with four pairs of caudal papillae (Fig. 4J-Q): one prominent pair in ventral preanal position, one pair in subventral adanal position, and two pairs at base of filiform tail appendage. The 3rd fused pair prominent in ventral position, the 4th intermediate-sized in subventral position. Long and narrow spicules present, fused at the middle. Gubernaculum absent or non-visible. Tail long, filiform.

### ***Cephalobellus potosiae*** Leibersperger, 1960

*Type specimens*: location unknown.

*Type host*: *Potosia cuprea* (Leibersperger, 1960),

*Current host: Cetonia* sp. (Scarabaeidae; Coleoptera).

*Type locality*: Germany (Leibersperger, 1960).

*Current locality*: Hungary

*Location in the host*: Intestine

*Prevalence and intensity:* average prevalence of 9%, two of the 10 investigated populations were infected; average intensity of 4.8 (4–5) (Table 1). Found with *C. cuspidatum* in one *Cetonia* sp. host, and with both *C. cuspidatum* and *C. osmodermae* in one *Melolontha* sp. individual.

*Material examined*: 7 female specimens, deposited in the invertebrate collection of the Swedish Museum of Natural History, Stockholm (slide accession numbers SMNH219633-SMNH219634).

### Redescription

**Fig. 5.**
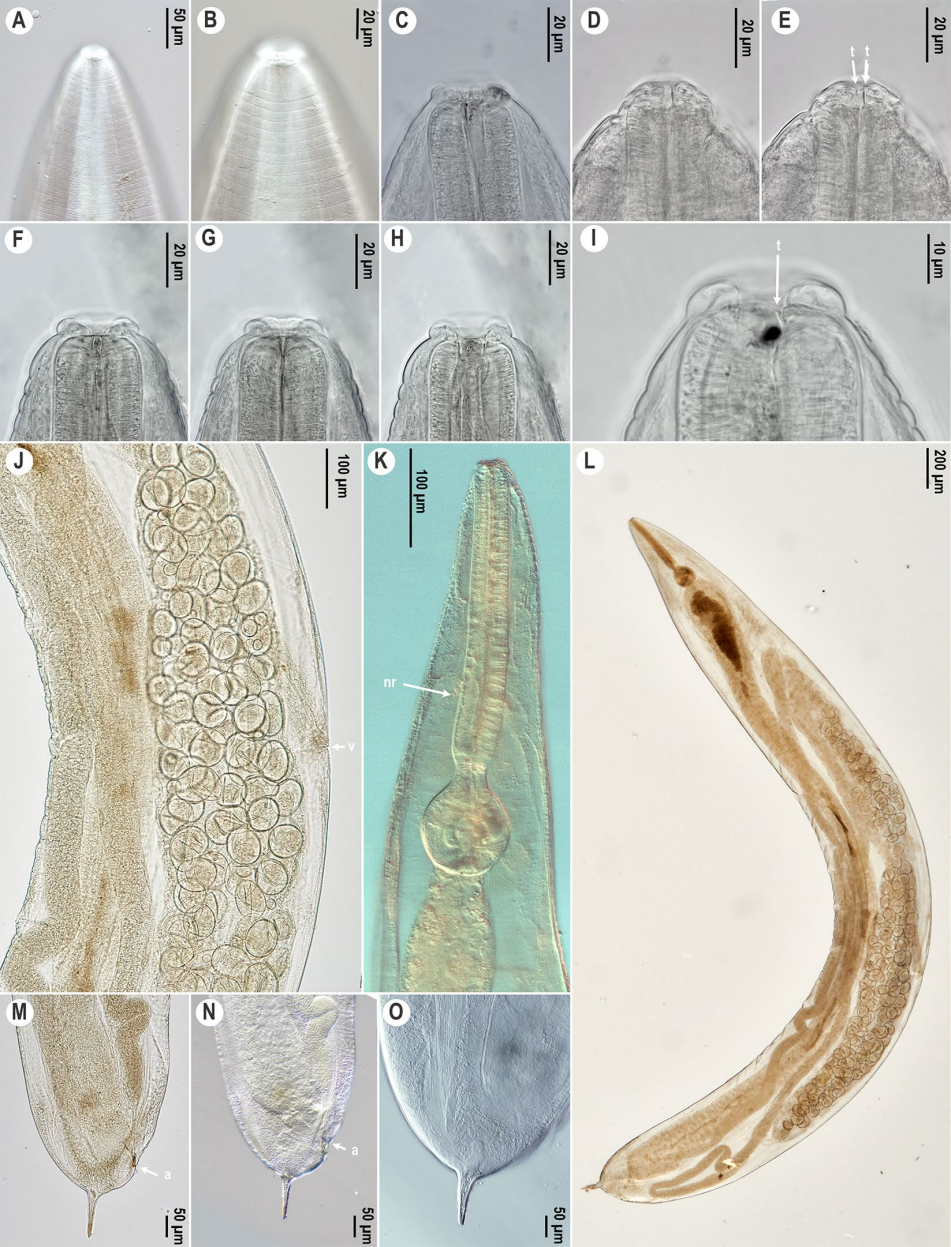
*Cephalobellus potosiae* Leibersperger, 1960 female: A-I, Anterior body end of different individuals in different focal planes; J, Vulval region, lateral view; K, Pharyngeal region, lateral view; L: Entire body, subventral view; M-O, Posterior end of different individuals (M-N, lateral view; O, dorsal view). *Abbreviations*: t: teeth; nr: nerve ring; v: vulva; a: anal opening.

*Female*. (Figure 5, Table 2) Long, spindle-shaped body, reaching maximum width at vulval region. Cuticle transversely striated and clearly annulated. Annulation detectable along entire body, weaker posterior to middle of body. Lateral ale absent. Labial annule set off from rest of body. First body annule longer than others, about 7 µm height, 34 µm width (Fig. 5A-H). Labial lobes not well-developed, eight fused labiopapillae present. Amphids not visible under the light microscope. Opening of low stoma triradiate, surrounded by hexagonal lining of cuticle. Two small teeth present in anterior part of te stoma (Fig. 5I). Excretory pore not visible in examined specimens under light microscope. Uterus containing numerous oval eggs (Fig. 5J). Eggs 1.5 times as long as wide. Egg shell without distinct ornamentation. Tail region dome-shaped ending in a short conical projection. Tail spike same length as dome (Fig. 5M-O). Phasmids not seen.

*Male.* Not found.

### Differences between studied specimens

The main characteristics distinguishing females of three studied species *C. cuspidatum, C. osmodermae* and *C. potosiae* is the proportion of two parts of the tail, the dome and the spike ratio, and to a lesser extent the size of the body (Table 2). Other differences between these species are the labial annuli and the vulval slit, which are not prominent in *C. cuspidatum* and *C. potosiae*, only in *C. osmodermae* specimens. Also, a pseudobulb (median bulb) is present in the corpus region of *C. osmodermae*.

The descriptions of *C. cuspidatum* (Rudolphi, 1814) and its synonyms, all published in the XIXth century, were quite brief and lacked detailed descriptions and illustrations. These old descriptions only briefly mentioned the morphology of the nematode corpus and the shape of the tail. In contrast, the description of *Thelastoma cuspidatum* (Rudolphi, 1814) by Théodoridès in 1955 included drawings of females, which closely resembled our specimens, except for the tail morphology. The drawings in Théodoridès' description did not depict phasmids, unlike what we observed in our specimens. Unfortunately, these descriptions did not provide measurements. However, we were able to obtain morphometric data for *C. cuspidatum* from the summary table provided by Jex et al. (2006b). Several measured features were different from the data in Jex et all.’s work: the the body length (2471–4578 µm in recent material instead of 5700 µm), the c-rate (14.4–22.1 in recent material instead 11.4) and the vulval body diameter (227–430 in recent material instead of 700 µm) (Jex et al., 2006b).

The original descriptions of *C. osmodermae* and *C. potosiae* included schematic drawings and some measurements but not thoroughly detailed descriptions or photographs (Leibersperger, 1960). Based on the original drawings, *C. osmodermae* females have two small teeth in the stoma. The description of *C. potosiae* did not include drawings of the stoma. In our results, two small teeth were present in *C. potosiae* stoma, but the teeth were absent in *C. osmodermae* individuals. Based on our observations, presence and location of the teeth in stoma is challenging to observe, primarily due to the dirt inside the stoma in majority of individuals. Among the measured features of *C. osmodermae,* only the maximum body width of type specimens was smaller than in our study (230–360 µm in recent material instead of 113–215 µm in the original description), but some of our specimens are obviously flattened. Several features are differed from the original descriptions of *C. potosiae*: a-ratio (9.6–16 in recent material instead of 19.5–36.8 in the original description), b-ratio (10.2–13.1 in recent material instead of 6.1–10.5 in the original description), T-ratio (44–49 in recent material instead of 53–57 in the original description), corpus length (236–354 µm in recent material instead of 370–520 µm in the original description), corpus diameter (40.6–54 µm in recent material instead of 24–42 µm in the original description), maximum body diameter (350–459 µm in recent material instead of 130–200 µm in the original description). On the other hand, this species is very variable in body size, e.g.: body length 2920–7360 µm, which is more than 4 mm range.

In this study, we found males only in *C. osmodermae*. Arrangement of papillae in males matches the original description and is different from any other known species of *Cephalobellus*.

### Molecular characterization and phylogenetic analysis

**Fig. 6.**
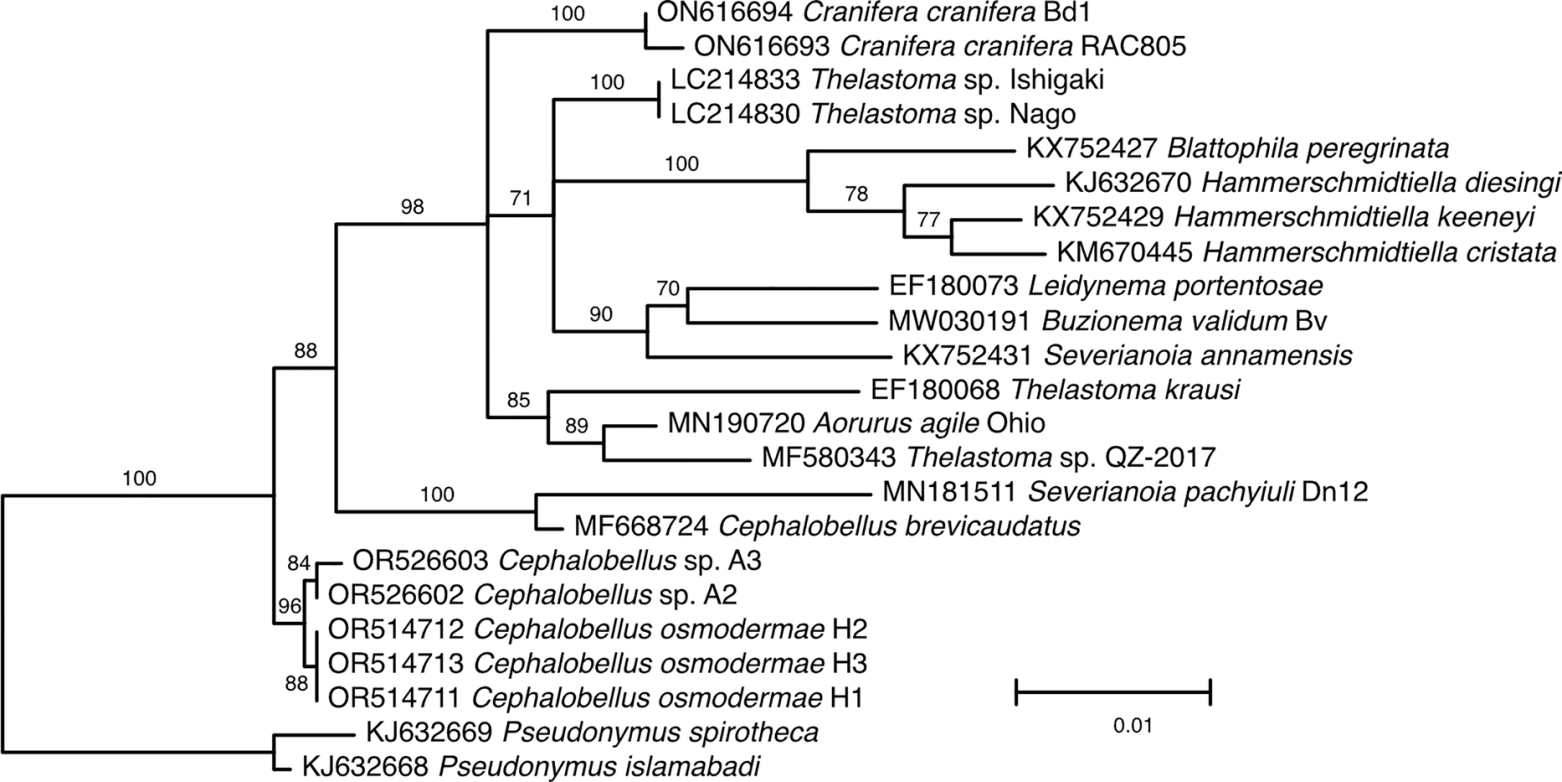
Maximum likelihood tree inferred using TIM2e+R2 model (rate parameters: A-C: 2.8229, A-G: 4.6281, A-T: 2.8229, C-G: 1.0000, C-T: 8.0524, G-T: 1.0000; state frequencies: equal) from alignment of nearly full length 18S rDNA showing relationships of *Cephalobellus osmodermae* Leibersperger, 1960 with other representatives of the family Thelastomatidae. The family Pseudonymidae is used as an outgroup.

**Fig. 7.**
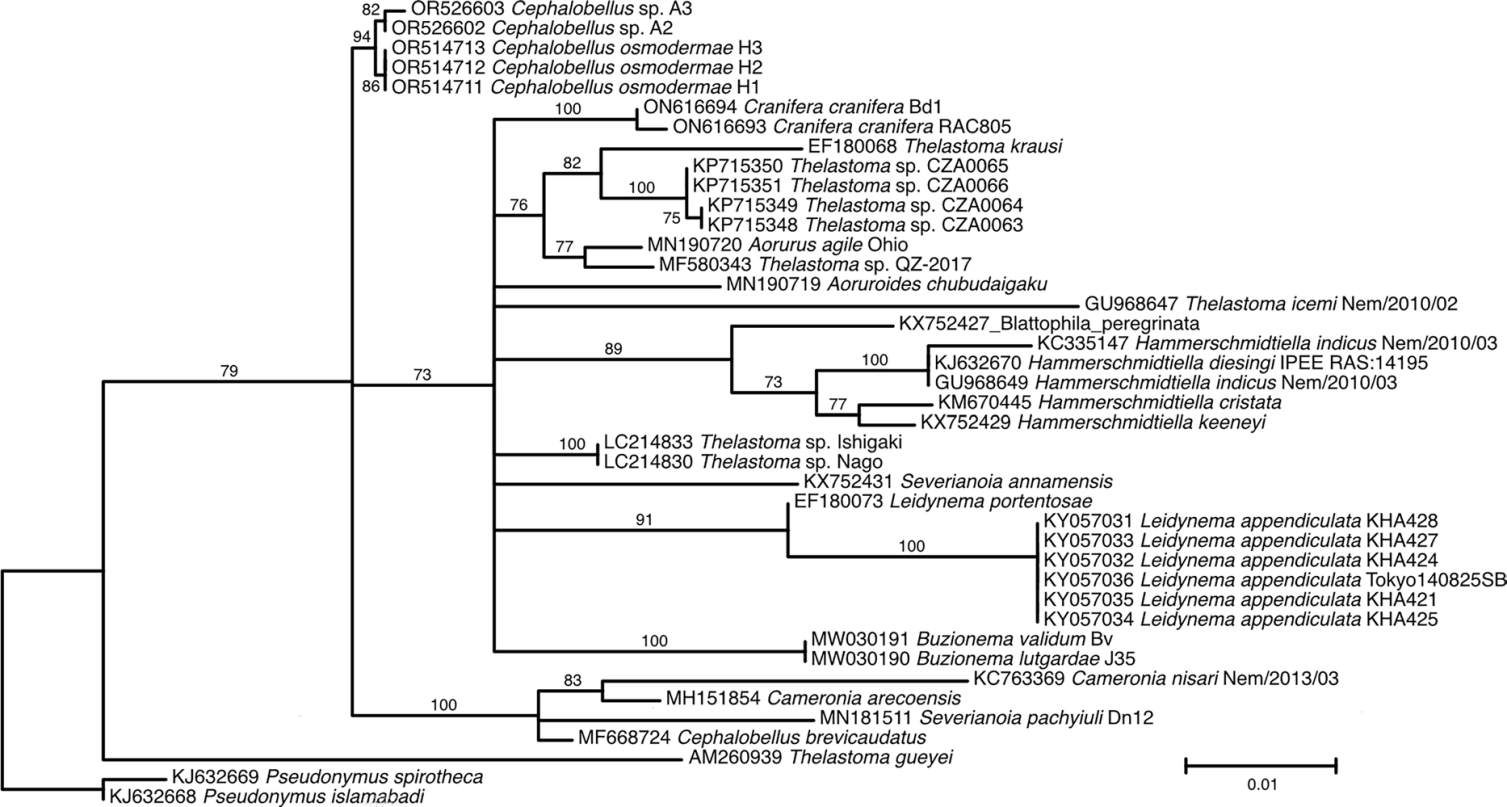
Maximum likelihood tree inferred using TIM2e+I+R2 (rate parameters: A-C: 2.5947; A-G: 3.8416; A-T: 2.5947; C-G: 1.0000; C-T: 7.6373; G-T: 1.0000; state frequencies: equal frequencies) from alignment of nearly full length and partial 18S rDNA showing relationships of *Cephalobellus osmodermae* Leibersperger, 1960 with other representatives of the family Thelastomatidae. The family Pseudonymidae is used as an outgroup.

**Fig. 8.**
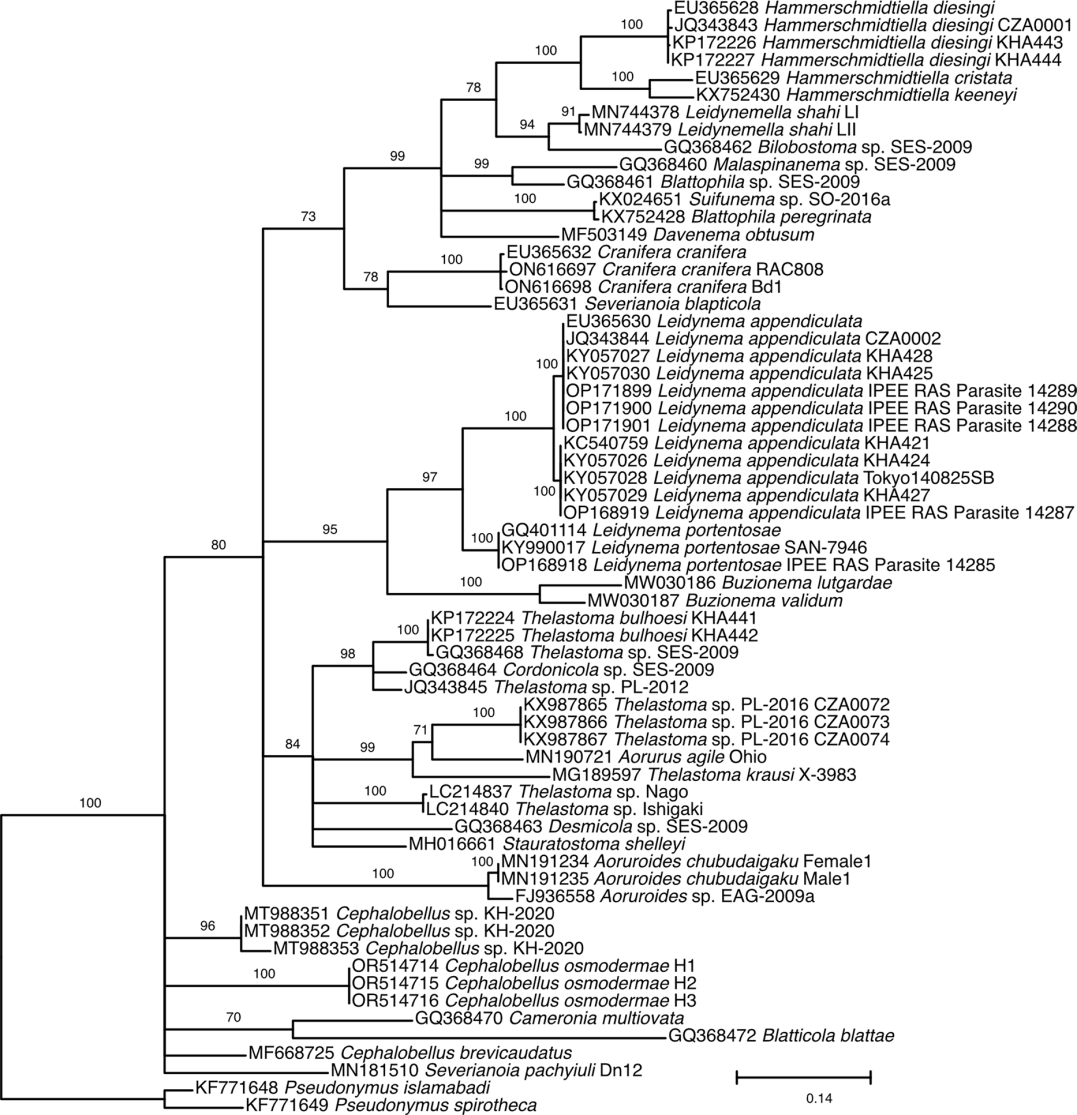
Maximum likelihood tree inferred using TIM3+F+I+G4 model (rate parameters: A-C: 0.6138, A-G: 3.8643, A-T: 1.0000, C-G: 0.6138, C-T: 6.1477, G-T: 1.0000; state frequencies: pi(A) = 0.2147, pi(C) = 0.213, pi(G) = 0.3175, pi(T) = 0.2549) from alignment of partial 28S rDNA showing relationships of *Cephalobellus osmodermae* Leibersperger, 1960 with other representatives of the family Thelastomatidae. The family Pseudonymidae is used as an outgroup.

**Fig. 9.**
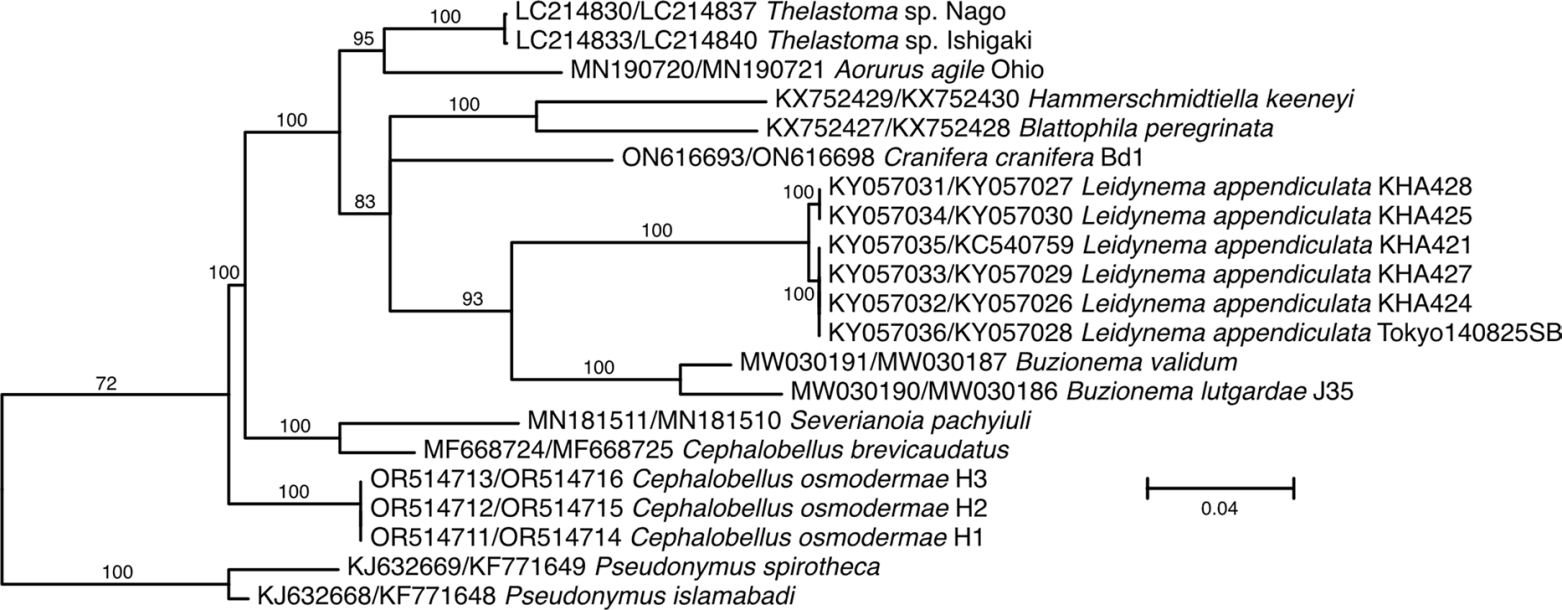
Maximum likelihood tree inferred using TIM3+F+I+G4 model (rate parameters: A-C: 0.4982; A-G: 3.1070; A-T: 1.0000; C-G: 0.4982; C-T: 5.0375; G-T: 1.0000; state frequencies: pi(A) = 0.2351; pi(C) = 0.2145; pi(G) = 0.2899; pi(T) = 0.2605) from concatenated alignment of partial 18S and 28S rDNA showing relationships of *Cephalobellus osmodermae* Leibersperger, 1960 with other representatives of the family Thelastomatidae. The family Pseudonymidae is used as an outgroup.

The phylogeny based on nearly full-length 18S rDNA sequences (Figure 6) is limited in a number of taxa available. All recently generated sequences form a monophyletic clade at the base of the phylogeny, but the genus *Cephalobellus* itself is paraphyletic, with *C. brevicaudatus* forming a monophyletic clade with *Severianoia pachyiuli* Malysheva, Shmatko & Spiridonov, 2020. Similarly, the genera *Severianoia* and *Thelastoma* are not monophyletic in this analysis. Adding partial 18S rDNA sequences to the analysis resulted in even less resolved phylogeny (Figure 7), where the position of *Thelastoma gueyei* does not receive any support, all recently generated sequences of *Cephalobellus* form a monophyletic clade as part of the basal trichotomy that also includes a monophyletic clade comprised of *Cephalobellus brevicaudatus* (Leidy, 1851) Christie, 1993, *Severianoa pachyiuli* and two species of *Cameronia* Basir, 1948, and a third clade leading to a large polytomy that includes all species of *Thelastoma* (polyphyletic) and *Severianoa annamensis* Luc & Spiridonov, 1993. The phylogenetic hypothesis based on partial 28S rDNA sequence (Figure 8) includes more species, but the tree itself is not well resolved. All three genera, *Cephalobellus, Severianoia* and *Thelastoma* are polyphyletic. However, most of *Thelastoma* samples included in the second analysis are not identified to species level, and not characterized morphologically, which makes it impossible to critically evaluate the topology of the phylogenetic tree. The concatenated analysis is most limited in the number of species included (Figure 9), and is thus the least informative in general, even though the tree topology is resolved well: the genus *Cephalobellus* is still resolved as polyphyletic, while the number of species of *Severianoia* (one) and *Thelastoma* (two unidentified) is too small to draw any conclusions.

### **Identification key to species of the genera*Cephalobellus, Severianoia*** and ***Thelastoma*found in scarab beetles**

1. Tail clearly conoid … 2

– Tail dome-shaped (cupola-shaped) with a spike of different lengths … 4

2. Female about 3 mm long, pharynx without pseudobulb … 3

– Female 6–9 mm long, pharynx with pseudobulb … ***Cephalobellus leukarti*** (Hammerschmidt, 1838), Christie, 1933 (host: *Melolontha aprilianus*, *Rhizotrogus solstitialis*; distribution: Germany)

3. Eggs nearly round; vagina/ovijector oblique, directed anteriad … ***Thelastoma brumpti*** Théodoridès, 1955 (host: *Glomeris annulata* and *Glomeris marginata* (Diplopoda), *Anomala dubia* var. *aenea* (Scarabaeidae); distribution France)

– Eggs elongate (1.5 times as long as they are wide), vagina/ovijector perpendicular to body axis … ***Severianoia brevicaudata*** Camino & Szathmary, 2001 (host: *Diloboderus abderus*; distribution: Argentina)

4. Spike equal to or shorter than the dome … 5

– Spike about 1.5–3 (rarely 4) times longer than the dome … 8

– Spike about 5–10 times longer than the dome … 9

5. Spike shorter than the dome, tail very short comparing to the nest of the body …

***Cephalobellus dollfusi*** Théodoridès, 1955 (host: *Anomala* sp.; distribution: France)

– Spike same length as the dome … 6

6. Distinct annulation along entire pharyngeal region … ***Cephalobellus potosiae*** Leibersperger, 1960 (host: *Potosia cuprea, Cetonia* sp.*, Melolontha* sp.; distribution: Germany, Hungary)

– Distinct annulation along 1/3 of the pharynx … 7

7. Nearctic, female body more than 3 mm long; b=9.5–9.7; c=23–24 … ***Cephalobellus cylindricum*** Christie, 1933 (host: unidentified Melolonthinae or Rutelinae; distribution: North America)

– Paleartic, female body less than 3 mm long; b=4.4–6; c=13.3–16 … ***Cephalobellus cetonicola* (**Théodoridès, 1955**) **Adamson and van Waerebeke, 1992 (host: *Cetonia* sp., *Potosia cuprea, Potosia* sp.; distribution: France)

8. Eggs 3 times as long as wide … ***Cephalobellus brevicaudatus*** (Leidy, 1851) Christie, 1993 (host: *Ligyrodes relictus, Tipula* (*Pterelachisus*) *trivittata*; distribution: Germany, France, North America (USA), North India, England)

– Eggs 1.5 times as long as wide … ***Cephalobellus cuspidatum* (**Rudolphi, 1814**) **Leibersperger, 1960 (host: *Oryctes nasicornis, Anomala dubia aenea, Anoxia* sp., *Cetonia aurata, Melolontha* sp.; distribution: Germany, France, Hungary)

9. Head with 8 distinct lobes … ***Thelastoma robustum*** Leidy, 1850 (host: *Osmoderma scabra, Xyloryctes satyrus*; distribution: USA)

– Head without developed lobes … 10

10. Excretory pore located posterior to bulb … ***Thelastoma gallicum*** Théodoridès, 1955 (host: *Oryctes nasicornis, Cetonia* sp., *Potosia cuprea, Potosia* sp.; distribution: France)

– Excretory pore located at the level of bulb or pharynx end … 11

11. Males with cloacal cone, females head with high stoma … 12

– Males without cloacal cone, females head with low stoma, pseudobulb present … ***Cephalobellus osmodermae*** Leibersperger, 1960 (host: *Osmoderma eremita, Potosia aeruginosa, Cetonia aurata, Melolontha* sp.; distribution: Germany, Hungary)

12. Palearctic species … ***Thelastoma gallicum*** Théodoridès, 1955 (host: *Oryctes nasicornis, Cetonia* sp., *Potosia cuprea, Potosia* sp.; distribution: France)

– Nearctic species … ***Thelastoma macramphidum*** Christie, 1931 (host: unidentified *Osmoderma* sp.; distribution: USA)

Footnotes: *Thelastoma brumpti* Théodoridès, 1955 is sometimes considered as a synonym of *Cephalobellus gallardi* (Dollfus, 1952) Basir, 1956. The species *C. galliardi* is a part of a species complex that also includes five other species, namely *C. lucani, C. lohmanderi, C. tipulae, C. glomeridis* and *C. granatensis* (Adamson and van Waerebeke, 1992). In present study we considered *Thelastoma brumpti* as valid species since it was isolated from a scarab beetle larva, while *C. galliardi* was found in a diplopod millipede, a notably distinct host. The revision of this species complex and the associated synonyms was conducted based on older literature sources.

## Discussion

The primary objective of our study was to investigate the presence of insect-associated nematode species within populations of agricultural pest beetles. Three pinworm species belonging to the genus *Cephalobellus* (Nematoda: Oxyuridomorpha: Thelastomatidae), namely *Cephalobellus cuspidatum, C. osmodermae*, and *C. potosiae*, were identified and redescribed in Hungary for the first time. These nematodes were extracted from the intestinal tracts of the larvae five populations each of scarab beetles European rose chafer beetle (*Cetonia aurata*) and cockchafer (*Melolontha* sp.) (Coleoptera: Scarabaeidae) from ten different localities in Hungary. A critical challenge in this study of thelastomatid pinworms was their identification based on morphology. Incomplete species descriptions and the absence of molecular reference data hampered the progress. The last major revision of Thelastomatidae dates back to 1992, and various authors have proposed contradictory lists of valid and invalid species, highlighting the urgent need for species redescriptions and clarification of their diagnostic features and limits or inter- and intraspecific variation. Our study contributes by providing detailed illustrations and descriptions of three *Cephalobellus* species. In addition, the newly generated sequences of *C. osmodermae* will serve as a reference for future molecular identifications and species differentiation within this genus.

*Cephalobellus potosiae* was exclusively found in two populations of *Melolontha* sp., while *C. cuspidatum* and *C. osmodermae* were present in both *Cetonia aurata* and *Melolontha* sp. Although these nematode species were not detected previously in either *Melolontha* sp. or *Cetonia* sp., it is known that host specificity is not very strict within different species of this genus. For example, *C. osmodermae* had been previously detected in two different beetle species belonging to the same family Scarabaeidae, *Osmoderma eremita* and *Potosia aeruginosa* (Leibersperger, 1960).

We observed instances of co-infection, where a single host was infected by multiple nematode species. *Cephalobellus cuspidatum* and *C. potosiae* were identified in single *Melolontha* sp. individual, and *C. osmodermae* with *C. cuspidatum* co-infected three *Cetonia aurata* host specimens. While other dual co-infections are documented in the literature (Ghosh, 2017; Nagae et al., 2021), our results represent a unique instance of a triple infection involving all three *Cephalobellus* species within a single *Melolontha* sp. individual. Furthermore, both *C. osmodermae* and *C. cuspidatum* co-infected with an insect-associated necromenic species, *Reiterina typica,* which was isolated from the host’s body coelom. The ecological relationship, evolutionary background, and the effect on the host of co-infecting nematode species are still unknown.

While our study primarily focused on the identification of nematode species in different hosts, the examination of more than 120 beetle larvae allowed us to establish the prevalence and the intensity of these pinworm species. *Cephalobellus osmodermae* showed the highest prevalence, particularly in the Budaörs locality, where all investigated grubs were infected by this species. Similarly, *Thelastoma krausi* Carreno, 2007, infected 100% of the hindgut of *Euryurus leachii* Gray, 1832 (Carreno, 2007). In other populations, the prevalence of *C. osmodermae* ranged from 9% to 53%. *Thelastoma vanwaerebekei* Orsini et al., 2018 had a slightly higher prevalence of 74% in *Gymnetis litigiosa* Gory & Percheron, 1833 scarab beetles (Orsini et al., 2018). *Cephalobellus cuspidatum* ranged from 17% to 65% prevalence, similar to *C. brevicaudatus*, which was found in 58.5% of *Tipula* sp. populations (Carreno et al., 2018). In contrast, *C. potosiae* infections were lower at 6% and 13% in the two infected populations. The intensity was the highest in *C. osmodermae*, while all investigated pinworm species exhibited a wide range of intensities, ranging from 2 to 45. Compared to other species, *C. brevicaudatus* showed an intensity of 3.2; *T. krausi* showed 3, and *T. vanwaerebekei* showed an intensity of 11.9 (Carreno, 2007; Carreno et al., 2018; Orsini et al., 2018).

In conclusion, this study represents a significant contribution to the understanding of the taxonomy and ecology of the genus *Cephalobellus* with detailed morphological redescriptions of the three species, along with the first-ever molecular data for *C. osmodermae.* While our phylogenetic analyses may not have yielded conclusive results, our research lays the foundation for future molecular identifications and species differentiation. To deepen our understanding of thelastomatid pinworms, future research should focus on the intricate aspects and host-parasite interactions, and co-infections exploring potential effects on the host organisms. Moreover, accurate morphological identification necessitates further species redescriptions and re-diagnoses, accompanied by comprehensive molecular data expanding into genomics.

## Data Availability

All studied specimens are deposited in permanent and accessible repositories: the invertebrate collection of the Swedish Museum of Natural History, Stockholm with slide accession numbers SMNH219625-SMNH219634. Sequences are deposited in GenBank with accession numbers OR514711-OR514716; OR526602-OR526603.

## References

[CR1] Adamson ML, van Waerebeke D (1992). Revision of the Thelastomatoidea, Oxyurida of invertebrate hosts I. Thelastomatidae. Systematic Parasitology.

[CR3] Andrássy, I., & Farkas, K. (1988). *Kertészeti növények fonálféreg kártevői. Agronematológiai kézikönyv.* Mezőgazdasági Kiadó.

[CR4] Balog, L. E., Holovachov, O., & Török, J. K. (2022a). Diversity of entomoparasitic nematodes in the rose chafer, *Cetonia aurata* grub. *7th International Congress of Nematology.* France, Antibes Juan-les-Pins.

[CR5] Balog, L. E., Holovachov, O., & Török, J. K. (2022b). Interactions between entomoparasitic nematodes within their host species, rose chafer grub. *7th International Congress of Nematology,* France, Antibes Juan-les-Pins.

[CR6] Balog LE, Török JK (2019). Parazita fonálférgek, mint a pajorok elleni lehetséges biokontroll eszközei. Növényvédelem.

[CR7] Bianchini, G. & Sánchez-Baracaldo, P. (2023). TreeViewer Version 2.1.0 (v2.1.0). *Zenodo*. 10.5281/zenodo.7768344

[CR8] Camino, N. B., & Reboredo, G. R. (2000). *Cephalobellus lobulat*a n. sp. (Oxyurida: Thelastomatidae) a Parasite of *Neocurtilla claraziana* Saussure (Orthoptera: Gryllotalpidae) from Argentina. *Memórias Do Instituto Oswaldo Cruz*, 95(1–2), 49–51. 10.1590/S0074-02762000000100007 10.1590/s0074-02762000000100007 10656704

[CR9] Camino NB, Reboredo GR (2005). A new Oxyurida (Thelastomatidae) from *Cyclocephala signaticollis burmeister* (Coleoptera: Scarabaeidae) from Argentina. Journal of Parasitology.

[CR10] Carreno, R. A. (2007). Description of a New Species of *Thelastoma* Leidy, 1849 (Nematoda: Oxyurida: Thelastomatidae) from the Millipede *Euryurus leachii* (Gray, 1832) in Ohio, U.S.A. *Comparative Parasitology*, 74(2), 211–217. 10.1654/4276.1

[CR11] Carreno RA (2014). The systematics and evolution of pinworms (Nematoda: Oxyurida: Thelastomatoidea) from invertebrates. Journal of Parasitology.

[CR12] Carreno, R. A., Kiebler, L., & Tuhela, L. (2018). First record of *Cephalobellus brevicaudatus* (Leidy, 1851) Christie, 1933 (Nematoda: Oxyurida: Thelastomatoidea), from *Cranefly larvae* (Diptera: Tipulidae) in Ohio, U.S.A. *Comparative Parasitology*, 85(2), 133–140. 10.1654/1525-2647-85.2.133

[CR13] Montes Garduño, de Oca U, Oceguera-Figueroa A (2020). Molecular Phylogeny of Thelastomatoidea (Nematoda) with the description of a new genus and two new species of *Hystrignathidae* associated with bess beetles (Coleoptera: Passalidae) from Oaxaca. Mexico. The Journal of Parasitology.

[CR14] Ghosh, J. (2017). A study on the occurrence of pinworms in the hindgut of *Periplaneta americana*. Journal of Parasitic Diseases, 41, 1153-1157. 10.1007/s12639-017-0952-0 10.1007/s12639-017-0952-0 PMC5660052 29114157

[CR15] Hodda, M. (2022). Phylum Nematoda: a classification, catalogue and index of valid genera, with a census of valid species. *Zootaxa*, 5114(1), 1-289. 10.11646/zootaxa.5114.1.1 10.11646/zootaxa.5114.1.1 35391386

[CR16] Holterman M, Van Der Wurff A, Van Den Elsen S, Van Megen H, Bongers T, Holovachov O, Bakker J, Helder J (2006). Phylum-wide analysis of SSU rDNA reveals deep phylogenetic relationships among nematodes and accelerated evolution toward crown clades. Molecular Biology and Evolution.

[CR17] Holterman M, Schratzberger M, Helder J (2019). Nematodes as evolutionary commuters between marine, freshwater and terrestrial habitats. Biological Journal of the Linnean Society.

[CR18] Huiting, H. F., Moraal, L. G., Griepink, F. C., & Ester, A. (2006). *Biology, control and luring of the cockchafer,* Melolontha melolontha. Wageningen, Applied Plant Research (Praktijkonderzoek Plant & Omgeving BV).

[CR19] Jex AR, Hu M, Rose HA, Schneider M, Cribb TH, Gasser RB (2006). Molecular characterization of Thelastomatoidea (Nematoda: Oxyurida) from cockroaches in Australia. Parasitology.

[CR20] Jex AR, Schneider MA, Rose HA, Cribb TH (2006). New thelastomatoidea (Nematoda: Oxyurida) from Australian burrowing cockroaches (Blaberidae: Geoscapheinae, Panesthiinae). Nematology.

[CR21] Kalyaanamoorthy S, Minh BQ, Wong TKF, Von Haeseler A, Jermiin LS (2017). ModelFinder: Fast Model Selection for Accurate Phylogenetic Estimates. Nature Methods.

[CR22] Larsson A (2014). AliView: a fast and lightweight alignment viewer and editor for large datasets. Bioinformatics.

[CR23] Leibersperger E (1960). Die Oxyuroidea der europaischen Arthropoden. Parasitologische Schriftenreihe.

[CR24] Minh BQ, Schmidt HA, Chernomor O, Schrempf D, Woodhams MD, Von Haeseler A, Lanfear R, Teeling E (2020). IQ-TREE 2: New Models and Efficient Methods for Phylogenetic Inference in the Genomic Era. Molecular Biology and Evolution.

[CR25] Nagae S, Sato K, Tanabe T, Hasegawa K (2021). Symbiosis of the millipede parasitic nematodes Rhigonematoidea and Thelastomatoidea with evolutionary different origins. BMC Ecology and Evolution.

[CR26] Nunn, G. B. (1992). *Nematode molecular evolution. Ph.D. Thesis.* University of Nottingham.

[CR27] Orsini, M. N.D., Cuellar, N., Rondan Dueñas, J. C., Gardenal, C. N., Doucet, M. E., & Lax, P. (2018). *Thelastoma vanwaerebekei* n. sp. (Oxyurida: Thelastomatidae) a parasite of *Gymnetis litigiosa* (Coleoptera: Scarabaeidae) from Uruguay. *Zootaxa*, 4375(1), 75–89. 10.11646/zootaxa.4375.1.3 10.11646/zootaxa.4375.1.3 29689780

[CR28] Rice P, Longden I, Bleasby A (2000). EMBOSS: the European Molecular Biology Open Software Suite. Trends in Genetics.

[CR29] Rudolphi CA (1814). Erster Nachtag zu meiner Naturgeschichte der Eingeweiderwurmer. Magazin fur die neusten Entdeckungenn in der gesammten Naturkunde. Gesellschaft Naturforschender Freunde Zu Berlin.

[CR30] Théodoridès J (1955). Contribution a l’étude des parasites et phorétiques de coléoptères terrestres. Vie Milieu Ser C Biol..

[CR31] Travassos L (1929). Contribuição preliminar á systematica dos nematodeos dos arthropodos. Memorias Do Instituto Oswaldo Cruz.

[CR32] Truett GE, Heeger P, Mynatt RL, Truett AA, Walker JA, Warman ML (2018). Preparation of PCR-quality mouse genomic DNA with hot sodium hydroxide and Tris (HotSHOT). Biotechniques.

[CR33] Zhang N, Yin S, Carreno RA, Zhang L (2021). Three new genera and new species of hystrignathid nematodes (Nematoda: Thelastomatoidea) from passalid beetles (Insecta: Passalidae) from Yunnan Province, China with phylogenetic analysis of Hystrignathidae. Zootaxa.

